# Microbial Flow Within an Air-Phyllosphere-Soil Continuum

**DOI:** 10.3389/fmicb.2020.615481

**Published:** 2021-01-12

**Authors:** Shu-Yi-Dan Zhou, Hu Li, Madeline Giles, Roy Neilson, Xiao-ru Yang, Jian-qiang Su

**Affiliations:** ^1^Key Laboratory of Urban Environment and Health, Institute of Urban Environment, Chinese Academy of Sciences, Xiamen, China; ^2^University of Chinese Academy of Sciences, Beijing, China; ^3^Center for Excellence in Regional Atmospheric Environment, Institute of Urban Environment, Chinese Academy of Sciences, Xiamen, China; ^4^Ecological Sciences, The James Hutton Institute, Dundee, United Kingdom

**Keywords:** phyllosphere, leaf microbiota, source tracking, microcosm, airborne microbial community

## Abstract

The phyllosphere is populated by numerous microorganisms. Microbes from the wider environment, i.e., air and soil, are considered key contributors to phyllosphere microbial communities, but their contribution is unclear. This study seeks to address this knowledge gap by controlling the movement of microbes along the air-phyllosphere-soil continuum. Customized equipment with dual chambers was constructed that permitted airflow to enter the first chamber while the second chamber recruited filtered microbe-free air from the initial chamber. *Allium schoenoprasum* (chive) and *Sonchus oleraceus* (sow thistle) were cultivated in both chambers, and the microbial communities from air, phyllosphere, and soil samples were characterized. Shares of microbial OTUs in the equipment suggested a potential interconnection between the air, phyllosphere, and soil system. Fast expectation-maximization microbial source tracking (FEAST) suggested that soil was the major source of airborne microbial communities. In contrast, the contribution of airborne and soil microbes to phyllosphere microbial communities of either *A. schoenoprasum* or *S. oleraceus* was limited. Notably, the soilborne microbes were the only environmental sources to phyllosphere in the second chamber and could affect the composition of phyllosphere microbiota indirectly by air flow. The current study demonstrated the possible sources of phyllosphere microbes by controlling external airborne microbes in a designed microcosm system and provided a potential strategy for recruitment for phyllosphere recruitment.

## Introduction

The phyllosphere represents one of the most important reservoirs of microorganisms on the planet (Lindow and Brandl, 2003; Monier and Lindow, 2004). On a global scale, it has been estimated that phyllosphere bacteria could comprise 10^26^ cells, with microbial density up to 10^6^–10^7^ cells per cm^2^ (Morris et al., 2002; Lindow and Brandl, 2003; Monier and Lindow, 2004). Phyllosphere bacteria are a component of the plant microbiome and have an important role in facilitating plant growth, protecting crops from external pathogens (Rasche et al., 2006), and mediating carbon and nitrogen cycles (Furnkranz et al., 2008; Redford and Fierer, 2009). Potential immigrants to the phyllosphere such as *Escherichia coli* pose a risk to human health through the transfer of human-pathogenic bacteria from ready-to-eat food (Teplitski et al., 2011; Chen et al., 2019; Zhou et al., 2020). Selection pressure is essential for structuring communities in the phyllosphere (Yang et al., 2001; Kim et al., 2012) and is typically strong as oligotrophic conditions provide low levels of nutrients in most part of the leaves (Lindow and Brandl, 2003). Additionally, the dynamic external environment such as fluctuations in UV light irradiation, humidity, and temperature is an additional pressure on phyllosphere microorganisms (Hirano and Upper, 2000; Kadivar and Stapleton, 2003; Redford and Fierer, 2009). Moreover, plant genotypes may contribute to the selection of phyllosphere microorganisms (Bodenhausen et al., 2014; Zarraonaindia et al., 2015; Agler et al., 2016).

The diversity of microorganisms within the phyllosphere is greater than previously thought but is lower than those in either rhizosphere or bulk soil (Delmotte et al., 2009; Knief et al., 2012). The phyllosphere microbial community does not exist in isolation and can recruit members from rainfall, irrigation, atmospheric deposition, and transfer from soils and other plant organs (Lopez-Velasco et al., 2013; Vacher et al., 2016). Soil has been widely considered a microbial reservoir for the plant microbiome (Vorholt, 2012; Gopal and Gupta, 2016). Emerging evidence suggests that soil microbial communities are important sources for the plant phyllosphere. For example, shared taxa have been found between the soil and the phyllosphere of grape, switchgrass, and perennial mustard, suggesting that soil is a potential contributor of phyllosphere microbes (Martins et al., 2013; Zarraonaindia et al., 2015; Grady et al., 2019). Furthermore, airborne microorganisms are another important source of phyllosphere microorganisms (Yan et al., 2020). Phyllosphere microbial communities also have the potential to affect the composition of the microbiome in surrounding air (Wei et al., 2017). In addition, the microbial composition of the phyllosphere could also be determined from seed hereditary (Kumar et al., 2005; Vacher et al., 2016). Furthermore, human intervention, such as irrigation and fertilization, could also be a driver of phyllosphere microbial communities (Zhu et al., 2017; Gekenidis et al., 2018). Meanwhile, human activities have the potential to introduce antibiotic-resistance genes and antibiotic-resistant pathogens and bacteria (ARB) into the plant system, posing a potential risk to human health (Chen et al., 2019).

While it is recognized that there are multiple sources to the phyllosphere microbial community including pathogenic bacteria or ARB, the contribution of each source is unknown (Vorholt, 2012). While previous studies have focused on the source-sink relationships between soil and phyllosphere or between air and phyllosphere (Lindemann and Upper, 1985; Martins et al., 2013), a more comprehensive evaluation is needed to understand the movement of bacteria within three sources (air, phyllosphere, and soil).

To determine the possible sources of phyllosphere bacteria, we designed a controlled system for plant cultivation that would prevent the recruitment of bacteria from external uncontrolled sources such as irrigation and aerosol deposition. By using a controlled environment, the external airborne microbes could be recruited as a potential source for the phyllosphere and at the same time facilitate evaluation of the contribution of possible other sources (air or soilborne microbes) to the phyllosphere. The second chamber used filtered air, enabling the evaluation of the contribution of soil solely. Thus, in this study we could characterize (1) the phyllosphere microbial community of two plant species and the impact of external airborne microbes to those phyllosphere communities and (2) determine the possible sources (air or soil-borne microbes) of the phyllosphere microbial communities.

## Materials and Methods

### Microcosm Design

**FIGURE 1:**
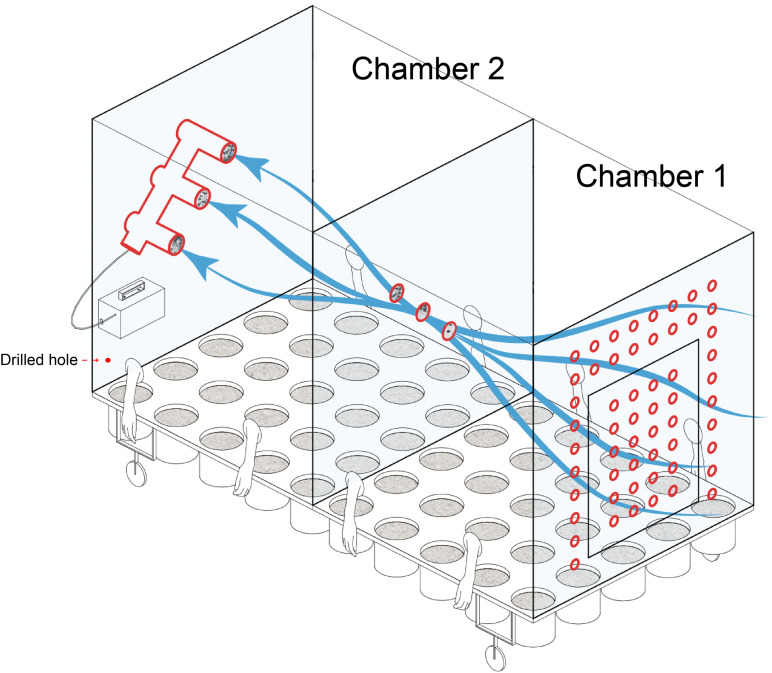
Schematic of the equipment used in the current study for plant cultivation and identifying the source of microbial communities.

A connected two-chamber device made of Acrylic plate was constructed for plant cultivation (Figure 1). One chamber permitted external air to flow through holes in one wall of the chamber, while the other chamber received filtered air from the previous chamber. A total of six filters (0.22 μm) with a diameter of 80 mm, located at the intersection of the two chambers and the back wall of chamber 2, were used for collection and filtering of airborne microbes. As chamber 1 had access to external air flow, it was considered for the purpose of this study as “microbial air” (M). As the air of chamber 2 was filtered, it was considered to be “non-microbial air” (NM).

A vacuum pump (flow rate was 200 L min^–1^) was used to induce the air flow within both chambers. To facilitate manipulation of plants while maintaining a sealed environment, sterile gloves were built into the chamber sides (Figure 1). Sterile water was used for irrigation to avoid the introduction of external microbes and entered through two drilled holes along each long chamber side. Built-in pots could be arranged in the culturing system. Except for the air holes in chamber 1, the system was hermetically sealed for the duration of the experiment. The specific design parameters are listed in supplementary materials (Supplementary Figure 1).

### Experimental Design and Sampling

Soil was collected from a vegetable field in Xiamen city, China (24°38′26.9″N 118°02′28.8″E) and sieved to 5 mm prior to use. *Allium schoenoprasum* and *Sonchus oleraceus* were chosen to evaluate the phyllosphere microbial community as they can be ingested raw as either a garnish or ready-to-eat salad and represent a major pathway for human health impact by phyllosphere microbiomes. Plants were cultivated in both chambers from seed and irrigated with sterile water. Plant pots and the surface of seeds were sterilized before cultivation. Plants were harvested after 30 days, and DNA was extracted from collected leaves. Airborne microbes were captured on filters (*n* = 6) for DNA extraction. Sample codes were the following: soils for cultivation of *A. schoenoprasum* and *S. oleraceus* were SCM and SSOM (in chamber 1), SCNM and SSONM (in chamber 2), respectively; the phyllosphere samples PCM and PSOM (in chamber 1) and PCNM and PSONM (in chamber 2); AM and ANM represented air samples with or without outdoor airborne microbes. Destructive sampling was conducted to collect the soil samples. Except for air samples (*n* = 3), there were four replicates for each sample type. All samples were stored at −20°C for DNA extraction.

### DNA Extraction From Soil, Phyllosphere, and Air Samples

The collection of microbes from leaves and subsequent DNA extraction was conducted as previously reported (Zhou et al., 2019). Collected soil samples were contained in a sterilized bag, and 500 mg soil was used for DNA extraction. Filters that captured airborne microbes from chambers 1 and 2 were cut into small pieces by sterile scissors prior to DNA extraction. DNA extractions of air, phyllosphere, and soil samples used a FastDNA Spin Kit for Soil (MP Bio, United States) following the manufacturer’s instructions. The quality of the extracted DNA was evaluated by a Qubit 3.0 Fluorometer (Invitrogen, Ghent, Belgium) (Zheng et al., 2020). Extracted DNA was stored at −20°C until used.

### Amplicon Sequencing of 16S RNA Genes and Analysis

The 515F/907R primer set was used to amplify the V4–V5 region of the 16S rRNA gene from air, phyllosphere, and soil samples (Turner et al., 1999). PCR protocols and conditions were as previously reported (Chen et al., 2017). To distinguish samples, unique barcodes were used for each sample. Prior to sequencing them on the Illumina 2,500 platform (Novogene, Beijing, China), the concentration of PCR products was quantified using a Qubit 3.0 Fluorometer. Quantitative Insight Into Microbiology Ecology (QIIME, version 1.9.1) was used for sequence analysis (Caporaso et al., 2010). Operational taxonomic units (OTU) were determined by UCLUST clustering (Edgar, 2010) with the similarity set at 97%. OTUs with only a single sequence were discarded from the final OTU table (Caporaso et al., 2010). Taxonomic classification of OTUs was performed using the RDP classifier with the Greengenes database version 13.5 (McDonald et al., 2012). Chao1, Observed species, PD whole tree, and Shannon diversity were calculated using QIIME. Sequences generated in this study were submitted to the National Center for Biotechnology Information (NCBI), with accession number PRJNA643678.

### Statistical Analysis

Excel 2016 was used for mathematical calculations of the raw data (for example, means, standard errors, and sum). Analysis of variance was conducted in SPSS 21, and *p* < 0.05 was considered statistically significant. Principal Coordinate Analysis (PCoA) based on Bray-Curtis distances and permutational multivariate analysis of variance (PERMANOVA) were performed using vegan package (Dixon, 2003; Oksanen et al., 2019) and visualized using ggplot2 package (version 3.1) (Wickham et al., 2020) in R. The microbial source was tracked using fast expectation-maximization microbial source tracking (FEAST), following protocols provided by the authors of the R package (Shenhav et al., 2019). Bar charts were created in OriginPro 2018.

## Results

### Composition of Microbial Communities

**FIGURE 2:**
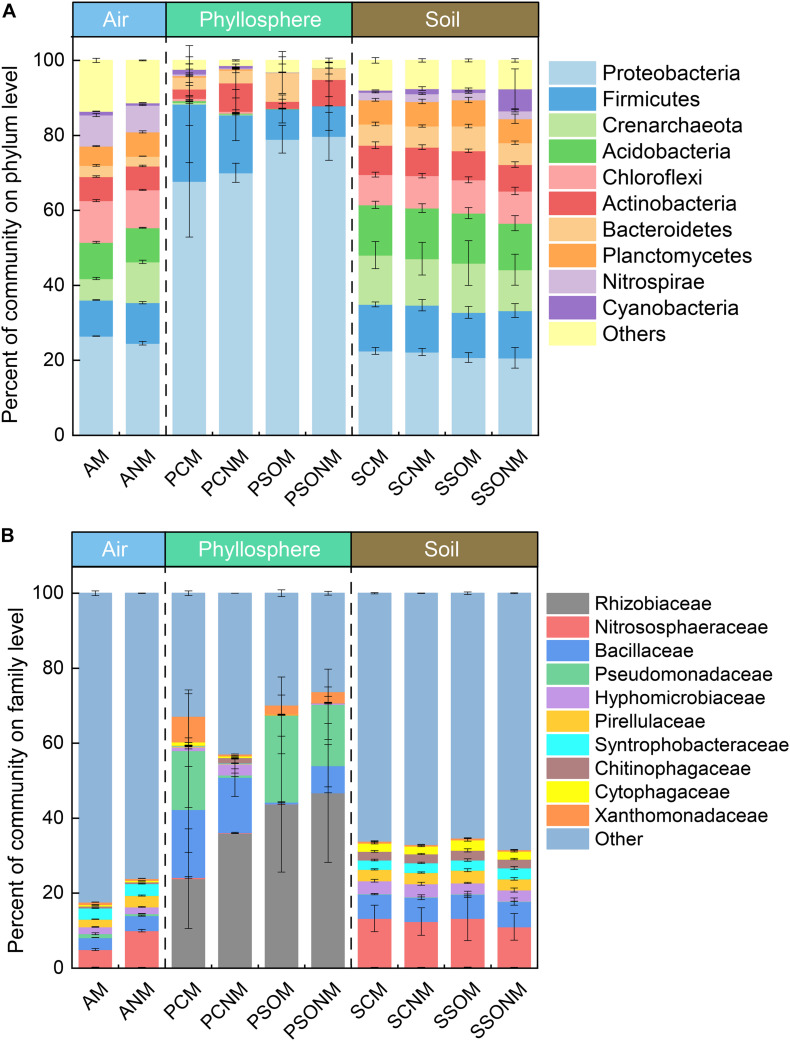
The percentage of microbial communities at phylum **(A)** and family **(B)** level, calculated by the average of replicates. “M” represents the chamber 1 samples (with extra outdoor microbes) and “NM” represents chamber 2 samples (without extra microbes). AM, PCM, PSOM, SCM, and SSOM represents the air, phyllosphere, and soil of *Allium schoenoprasum* and *Sonchus oleraceus* samples, respectively, in chamber 1 (with external airborne microbes). The ANM, PCNM, PSONM, SCNM, and SSONM represents the air, phyllosphere, and soil of *A. schoenoprasum* and *S. oleraceus* samples, respectively, in chamber 2 (without external airborne microbes).

A total of 3,947,497 high-quality sequences, which ranged from 48,053 (air) to 245,792 (soil) were recorded across all samples. Proteobacteria (25.5%), Chloroflexi (10.6%), Acidobacteria (9.3%), Firmicutes (7.7%), and Nitrospirae (7.5%) made up more than 65% of airborne microbial OTUs (Figure 2A). In contrast, Proteobacteria (74.8%) dominated phyllosphere samples, followed by Firmicutes (12.4%), Bacteroidetes (4.7%), and Actinobacteria (4.3%). Compared with both air and phyllosphere microbial communities, soil microbial communities were relatively homogeneous with the similar relative abundance of Proteobacteria, Acidobacteria, Firmicutes, and Crenarchaeota across all soil samples. The relative abundance of Proteobacteria in the phyllosphere was significantly greater than that in either air or soil samples (*p* < 0.01, ANOVA). Accordingly, the relative abundance of Acidobacteria, Chloroflexi, Planctomycetes, Nitrospirae, and Crenarchaeota in the phyllosphere were significantly lower than those in air and soil samples (*p* < 0.05, ANOVA). ANOVA results at phylum level are presented in the supplementary materials (Supplementary Tables 1, 2).

The dominant families with relative abundance >1% in were Nitrososphaeraceae (7.3%), Bacillaceae (3.6%), Syntrophobacteraceae (3.1%), Pirellulaceae (2.6%), and Hyphomicrobiaceae (1.8%) (Figure 2B) in air samples. Rhizobiaceae (38.2%), Pseudomonadaceae (15.8%), and Bacillaceae (9.0%) represented 63.3% of the total microbial community in the phyllosphere. The abundance of Rhizobiaceae and Pseudomonadaceae was significantly greater in phyllosphere (*p* < 0.05, ANOVA) than either air or soil samples (Supplementary Tables 3, 4).

### Comparative Analysis of Air, Phyllosphere and Soil Microbial Communities

**FIGURE 3:**
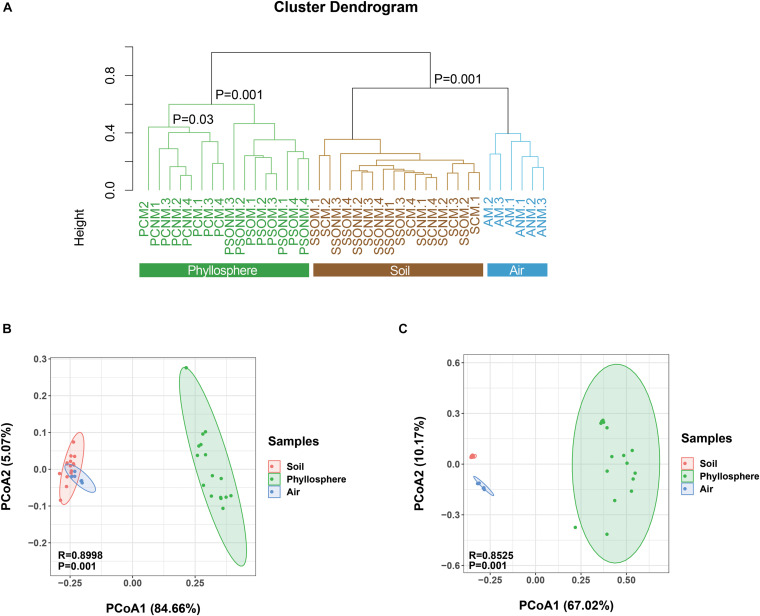
Dissimilarity analysis of microbial OTUs, phyla, and families for all samples. “M” represents chamber 1 samples (with extra outdoor microbes) and “NM” represents chamber 2 samples (without extra microbes). **(A)** Cluster diagram at OTU level. **(B,C)** The Principal Coordinate Analysis (PCoA) is based on the Bray-Curtis distance for microbial phylum and family, respectively. Different colors represent different habitats, while different shapes indicate each chamber.

The diversity of microbial communities in the phyllosphere was significantly lower than those of air and soil samples (*p* < 0.05, ANOVA, Supplementary Figure 2). Average linkage clustering of OTU level data (Figure 3A) revealed a clear separation between air, phyllosphere, and soil samples (*p* = 0.001, ANOVA). The different plant species, *Sonchus oleraceus* and *A. schoenoprasum*, separated into different clusters (*p* = 0.001, PERMANOVA). The phyllosphere samples of *A. schoenoprasum* from different chambers (with or without external air microbiota) were separated into two clusters; however, the phyllosphere samples of *S. oleraceus* were clustered together (Figure 3A).

At the phylum level, the composition of microbes from air, phyllosphere, and soil samples clearly separated along PCo1 (*p* < 0.001, PERMANOVA, Figure 3B), which explained 84.7% of the total variance. At the family level, a similarly significant separation existed along PCo1, explaining 67.0% of the total variance (Figure 3C). The phyllosphere microbial communities were significantly different between the two plant species in both chambers 1 and 2 (*p* < 0.001, PERMANOVA, Supplementary Figures 3A,B). Within the same plant species, the *A. schoenoprasum* phyllosphere microbial communities were separated between chamber 1 and 2 along the PCo1 which explained 42% of the variance (*p* < 0.05, PERMANOVA, Supplementary Figure 3C). In contrast, no significant differences were observed within *S. oleraceus* samples (*p* > 0.05, PERMANOVA, Supplementary Figure 3D).

### The Shared Microbiota and Source Tracking

**FIGURE 4:**
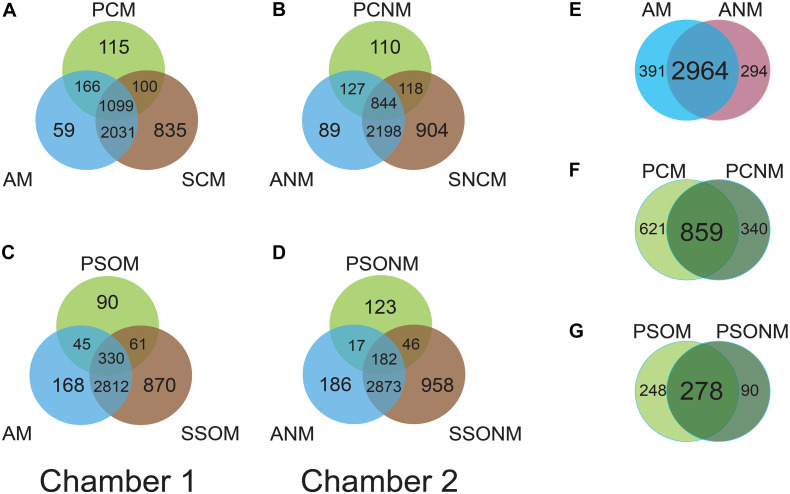
Venn diagram at OTU level for the two chambers. “M” represents chamber 1 samples (with extra outdoor microbes) and “NM” represents chamber 2 samples (without extra microbes). AM and ANM, PCN and PCNM, PSOM and PSONM represent the air, phyllosphere, and soil samples of *Allium schoenoprasum* and *Sonchus oleraceus* in chambers 1 and 2 (with or without extra airborne microbes), respectively. **(A,C)** represent the overview of shared OTUs between air, phyllosphere, and soil samples in chamber 1; **(B,D)** represent shared OTUs between air, phyllosphere, and soil samples in chamber 2. **(E–G)** represent shared microbial OTUs between AM and ANM, PCN and PCNM, PSOM and PSONM, respectively.

The shared microbiota among air, phyllosphere, and soil samples in each chamber were analyzed at the OTU level. In chamber 1, air (AM), *A. schoenoprasum* phyllosphere (PCM) and *A. schoenoprasum* soil (SCM) samples shared 1099 OTUs. AM and SCM shared 3130 OTUs, which was greater than those between either AM and PCM (1265) or PCM and SCM (1199). A total of 115 and 90 unique microbial OTUs were found in the phyllosphere of *A. schoenoprasum* (PCM) and *S. oleraceus* (PSOM), respectively (Figures 4A,C). A similar pattern of shared OTUs was observed in chamber 2 (Figures 4B,D). 391 (chamber 1) and 294 (chamber 2) unique OTUs were found in air samples, which represents 10.7% and 8.1% of the total OTUs, respectively (Figure 4E). There were 2964 OTUs were shared between AM and ANM, which accounted for 88.3% and 91.0% of the total OTUs in each chamber. The PCN and PCNM, PSOM and PSONM shared a total 859 and 278 OTUs in chambers 1 and 2, respectively (Figures 4F,G).

**FIGURE 5:**
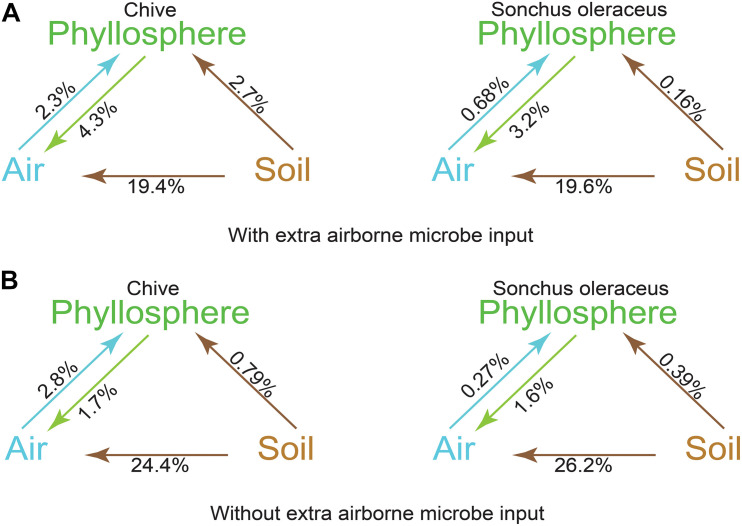
Fast expectation-maximization microbial source tracking (FEAST) analysis for chamber 1 **(A)** and chamber 2 **(B)** based on OTUs level. Direction of the arrows represents the source-sink relationships, and percentages represent the contribution that each source provides.

A source tracking method, FEAST, was used for tracking the origin of the phyllosphere microbiota based on the OTU data. Contributions from each source to sink were calculated and represented as a percentage. The airborne microbial community had approximately 19.5% of the community sourced from soil and 3% (*S. oleraceus*) and 4% (*A. schoenoprasum*) from the respective phyllosphere (Figure 5A). A total of 5.0% of the microbiota in *A. schoenoprasum* phyllosphere originated from the air (2.3%) and *A. schoenoprasum* soil (2.7%). Soil associated with *S. oleraceus* and the air each accounted for <1% of the *S. oleraceus* phyllosphere communities. In general, the relative abundance of each microbial source to each of the three (air, phyllosphere, and soil) microbial communities in chamber 2 was similar to that for chamber 1 (Figure 5B).

## Discussion

### The Composition of Microbes

In this study, we created a purpose-built system that controlled air movement for comparison of microbial communities between air, phyllosphere, and soil samples. Air from the external environment entered chamber 1 and then chamber 2 after filtration. Results demonstrated that the composition of the microbial communities was significantly different in the three compartments (*p* < 0.05, PERMANOVA), which is consistent with previous studies (Vokou et al., 2012; Bulgarelli et al., 2013). At the phylum level, the archaea *Crenarchaeota* was sharply lower (<1%) in the phyllosphere but higher in both air and soil samples, which concurred with the previous study (Vorholt, 2012). The shared of *Crenarchaeota* within air, phyllosphere, and soil suggested that soil may be a possible source of archaea affecting adjacent air (Wehking et al., 2018). Previous studies have found that the bacterial families of *Rhizobiaceae* and *Pseudomonadaceae* are commonly observed in plants, which would help create differences in composition of the phyllosphere, air, and soil samples (Yanni et al., 1997; Hunter et al., 2010). A significantly higher abundance (*p* < 0.001, ANOVA) of *Rhizobiaceae* in the phyllosphere than the other habitats, which indicated that the phyllosphere may be the ideal habitat for surviving of *Rhizobiaceae*. It has been reported that the Rhizobiaceae can migrate from root to the above-ground plant parts through endophytic system in rice or tobacco, which suggested that phyllosphere may acquire certain microbes from soil and finally to the leaves (Chi et al., 2005; Ji et al., 2010).

The composition of soil microbial communities between the two chambers was similar, and there was no difference in the soil microbes associated with the two plant species used in this study. Although the same soil was used for growing both plant species, the phyllosphere microbial community composition was clearly distinct for each of the two plant species in both chambers. For instance, the phyla of *Acidobacteria*, *Crenarchaeota*, and *Chloroflexi* were significantly higher in PCM than PSOM in chamber 1. *Hyphomicrobiaceae*, *Pirellulaceae*, and *Syntrophobacteraceae* were significantly higher in PCNM than PSONM in chamber 2, which suggested plant species may therefore be one of the drivers to shape the microbial community in the phyllosphere (Whipps et al., 2008; Redford et al., 2010).

The differentiated composition of dominant taxa between the phyllosphere and other habitats suggested that rare species from the soil and air may colonize and grow on plant leaves then become dominant taxa in phyllosphere communities. The depth of the sequencing may be insufficient to effectively characterize rare species in microbial communities in air, phyllosphere, and soil sample.

### Source of the Phyllosphere Microbial Community

As environmental factors were controlled in a sealed system in this study, FEAST analysis has the potential to identify the sources of the microbial communities in the air and phyllosphere. Unlike with previous studies, in this system we can precisely identify the source of the microbial communities in the air, phyllosphere, and soil samples (Zarraonaindia et al., 2015; Chen et al., 2017). In addition, the microcosm system allowed us to evaluate the contribution of soil solely by excluding the airborne microbes in chamber 2.

To understand the interactions between the air, phyllosphere, and soil samples, shared OTUs were investigated. The number of shared microbial OTUs between air and soil as well as between phyllosphere and soil samples was considerable, which suggested a potential interconnection of microbes from these sources. The number of unique OTUs in AM (391) and ANM (294) were fewer than those shared between AM and ANM (2,964). This difference in OTU number between air samples may have been affected by the microbial composition of soil and phyllosphere sources in each chamber. As the air was continuously in motion, the communities in the soil and phyllosphere may have been distributed into the air within each chamber, as perhaps highlighted with the noted contribution of phyllosphere and soil microbes to the air microbial communities (Lindemann and Upper, 1985; Lymperopoulou et al., 2016). Soil and phyllosphere microbes were the main sources of airborne microbial communities in both chambers. It is likely that the air circulation in the chamber may have resulted in the suspension of soil and phyllosphere microbes in the air, allowing them to become part of the airborne microbial community (Bowers et al., 2013; Smets et al., 2016). To judge from this system, the contribution of the phyllosphere to the airborne microbial community was greater than that from the air to the phyllosphere, which concurred with Lymperopoulou et al. (2016).

Soil can affect the microbial communities in the phyllosphere either directly or indirectly. It has been reported that microbes originating from the soil can colonize plant roots and then reach the phyllosphere (Beattie and Lindow, 1999; Bodenhausen et al., 2013) through endophytic transport (Chi et al., 2005), which would directly affect the phyllosphere microbiome. Previous studies had been mainly focused on this direct effect. However, there are few studies that consider the indirect effect of the soil microbial communities on the phyllosphere, such as through airborne distribution. In the system, the composition of airborne microbes in chamber 2 was predominately acquired from soil microbes through air currents with no external microbes input from environment, which implied a potential pathway for microbes transportation from soil to air and finally to phyllosphere (Lindemann and Upper, 1985; Martins et al., 2013; Grady et al., 2019). Thereby, soil microbes may indirectly contribute to phyllosphere microbes. In this study, the contributions of the soil and air to the phyllosphere microbial communities were low, whereas a considerable proportion of unknown sources contributed to the phyllosphere microbial communities according to FEAST analysis. Although there was a shared component of the microbial community in the soil, air, and phyllosphere, none of the shared microbes were dominant microbes, which suggests that plant microbes may be acquired inherently from seed (Wulff et al., 2003; Vorholt, 2012). The results from a recent study (preprint) supports our point (Abdelfattah et al., 2020). Thus, inheritance of microbes first from seeds and parent material and supplemented from the surrounding environment, such as the soil and air, may be the potential pathway to explain the origination of phyllosphere microbes (Mukhopadhyay et al., 1996; Truyens et al., 2015; Cope-Selby et al., 2017). Future studies including the seed microbiome with a time-series sampling strategy are needed for more effective characterization of community change in each habitat.

In summary, the identified shared taxa highlighted the potential exchange of microbes within the air-phyllosphere-soil continuum. Part of the airborne microbes originated from the soil and phyllosphere in both chambers. However, source tracking analysis indicated the soil and air may not be the major sources of the *A. schoenoprasum* and *S. oleraceus* phyllosphere microbial communities, although we found considerable taxa overlap within three habitats.

## Data Availability Statement

The datasets presented in this study can be found in online repositories. The names of the repository/repositories and accession number(s) can be found in the article/Supplementary Material.

## Author Contributions

S-Y-DZ contributed to the conception, analysis, depict, and drafted the manuscript. HL contributed to the conception, design of the microcosm, experiment, and review of the manuscript. MG, RN, X-rY, and J-qS contributed to the review of the manuscript. All authors gave the final approval and agreed to be accountable for all aspects of the work.

## Conflict of Interest

The authors declare that the research was conducted in the absence of any commercial or financial relationships that could be construed as a potential conflict of interest. The reviewer DJ declared a shared affiliation with several of the authors S-Y-DZ, HL, and X-rY to the handling editor at the time of review.

## Supplementary Material

Supplementary Figure 1 The specific design parameters of the equipment.

Supplementary Figure 2 Chao1, Observed species, PD whole tree, and Shannon analysis of Alpha diversity.

Supplementary Figure 3 Principal Coordinate Analysis (PCoA) based on Bray-Curtis distances of microbial OTU.
