# Supplementary material for: Microbial Flow Within an Air-Phyllosphere-Soil Continuum
Source: Front Microbiol. 2021 Jan 12;11:615481. doi: 10.3389/fmicb.2020.615481 (PMC7873851; doi:10.3389/fmicb.2020.615481)
Supplement: Supplementary Figure 1 — The specific design parameters of the equipment. [file Data_Sheet_1.docx]

Microbial flow within an air-phyllosphere-soil continuum

Shu-Yi-Dan Zhou ^1, 2, a^, Hu Li ^1, 2, 3, a,^ *, Madeline Giles ^4^, Roy Neilson ^4^, Xiao-ru Yang ^1, 2, 3^, Jian-qiang Su ^1, 2, 3^

*^1^ Key Laboratory of Urban Environment and Health, Institute of Urban Environment, Chinese Academy of Sciences, 1799 Jimei Road, Xiamen 361021, China.*

*^2^ University of Chinese Academy of Sciences, 19A Yuquan Road, Beijing 100049, China.*

*^3^ Center for Excellence in Regional Atmospheric Environment, Institute of Urban Environment, Chinese Academy of Sciences, Xiamen 361021, China*

*^4^ Ecological Sciences, The James Hutton Institute, Dundee, DD2 5DA, Scotland, UK*

**Corresponding Author**

* Corresponding author: Hu Li

Key Laboratory of Urban Environment and Health, Institute of Urban Environment, Chinese Academy of Sciences, 1799 Jimei Road, Xiamen 361021, China

E-mail address: [hli@iue.ac.cn](mailto:hli@iue.ac.cn) (H. Li)

**
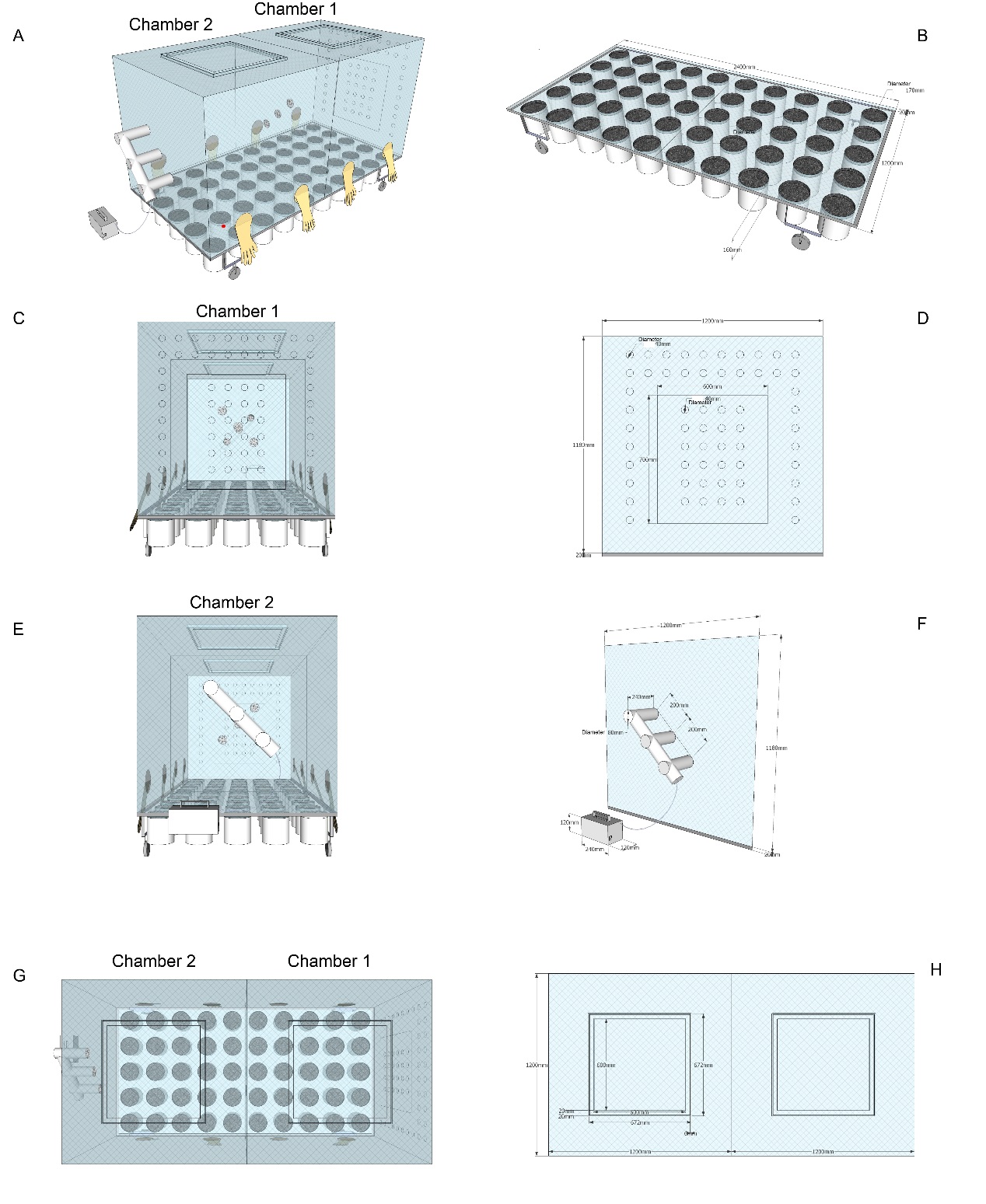
**

**Figure S1.** Design parameters of the constructed chambers used in this study. (A) and (B) front view of the equipment. (C), (D), (E) and (F) represent the right and left elevations of the equipment. (G) and (H) top view of the equipment.

Table S1. The analysis of variance among soil, phyllosphere and air samples (phylum level).

|  | | | Mean Difference (I-J) | Std.Error | Sig. | 95% Confidence Interval | |
| --- | --- | --- | --- | --- | --- | --- | --- |
|  |  |  |  |  |  | Lower Bound | Upper Bound |
|  |  |  |  |  |  |  |  |
| Proteobacteria | Soil | Phyllosphere | -.52515^*^ | .02212 | .000 | -.5701 | -.4802 |
|  |  | Air | -.03978 | .02995 | .193 | -.1006 | .0210 |
|  | Phyllosphere | Soil | .52515^*^ | .02212 | .000 | .4802 | .5701 |
|  |  | Air | .48538^*^ | .02995 | .000 | .4246 | .5462 |
| Firmicutes | Soil | Phyllosphere | -.00873 | .02324 | .709 | -.0559 | .0385 |
|  |  | Air | .02171 | .03147 | .495 | -.0422 | .0856 |
|  | Phyllosphere | Soil | .00873 | .02324 | .709 | -.0385 | .0559 |
|  |  | Air | .03044 | .03147 | .340 | -.0335 | .0943 |
| Acidobacteria | Soil | Phyllosphere | .12971^*^ | .00346 | .000 | .1227 | .1367 |
|  |  | Air | .03777^*^ | .00468 | .000 | .0283 | .0473 |
|  | Phyllosphere | Soil | -.12971^*^ | .00346 | .000 | -.1367 | -.1227 |
|  |  | Air | -.09193^*^ | .00468 | .000 | -.1014 | -.0824 |
| Crenarchaeota | Soil | Phyllosphere | .12227^*^ | .01130 | .000 | .0993 | .1452 |
|  |  | Air | .04090^*^ | .01530 | .011 | .0098 | .0720 |
|  | Phyllosphere | Soil | -.12227^*^ | .01130 | .000 | -.1452 | -.0993 |
|  |  | Air | -.08137^*^ | .01530 | .000 | -.1124 | -.0503 |
| Actinobacteria | Soil | Phyllosphere | .02941^*^ | .00871 | .002 | .0117 | .0471 |
|  |  | Air | .01170 | .01179 | .328 | -.0122 | .0356 |
|  | Phyllosphere | Soil | -.02941^*^ | .00871 | .002 | -.0471 | -.0117 |
|  |  | Air | -.01771 | .01179 | .142 | -.0416 | .0062 |
| Chloroflexi | Soil | Phyllosphere | .08282^*^ | .00217 | .000 | .0784 | .0872 |
|  |  | Air | -.02065^*^ | .00294 | .000 | -.0266 | -.0147 |
|  | Phyllosphere | Soil | -.08282^*^ | .00217 | .000 | -.0872 | -.0784 |
|  |  | Air | -.10347^*^ | .00294 | .000 | -.1094 | -.0975 |
| Bacteroidetes | Soil | Phyllosphere | .01593 | .00874 | .077 | -.0018 | .0337 |
|  |  | Air | .03132^*^ | .01183 | .012 | .0073 | .0553 |
|  | Phyllosphere | Soil | -.01593 | .00874 | .077 | -.0337 | .0018 |
|  |  | Air | .01539 | .01183 | .202 | -.0086 | .0394 |
| Planctomycetes | Soil | Phyllosphere | .06382^*^ | .00212 | .000 | .0595 | .0681 |
|  |  | Air | .00759^*^ | .00288 | .012 | .0017 | .0134 |
|  | Phyllosphere | Soil | -.06382^*^ | .00212 | .000 | -.0681 | -.0595 |
|  |  | Air | -.05623^*^ | .00288 | .000 | -.0621 | -.0504 |
| Nitrospirae | Soil | Phyllosphere | .01882^*^ | .00122 | .000 | .0163 | .0213 |
|  |  | Air | -.05669^*^ | .00165 | .000 | -.0600 | -.0533 |
|  | Phyllosphere | Soil | -.01882^*^ | .00122 | .000 | -.0213 | -.0163 |
|  |  | Air | -.07551^*^ | .00165 | .000 | -.0789 | -.0722 |
| Cyanobacteria | Soil | Phyllosphere | .01666^*^ | .00783 | .041 | .0008 | .0326 |
|  |  | Air | .01399 | .01061 | .196 | -.0075 | .0355 |
|  | Phyllosphere | Soil | -.01666^*^ | .00783 | .041 | -.0326 | -.0008 |
|  |  | Air | -.00267 | .01061 | .803 | -.0242 | .0189 |
| Other | Soil | Phyllosphere | .01744^*^ | .00259 | .000 | .0122 | .0227 |
|  |  | Air | -.00882^*^ | .00351 | .017 | -.0159 | -.0017 |
|  | Phyllosphere | Soil | -.01744^*^ | .00259 | .000 | -.0227 | -.0122 |
|  |  | Air | -.02625^*^ | .00351 | .000 | -.0334 | -.0191 |
| *. The mean difference is significant at the 0.05 level. | | | | | | | |

Table S2. The analysis of variance between plant species in different chamber (phylum level).

|  | | | Mean Difference (I-J) | Std.Error | Sig. | 95% Confidence Interval | |
| --- | --- | --- | --- | --- | --- | --- | --- |
|  |  |  |  |  |  | Lower Bound | Upper Bound |
|  |  |  |  |  |  |  |  |
| Proteobacteria | PSONM | PSOM | .00777 | .05973 | .899 | -.1224 | .1379 |
|  |  | PCNM | .10045 | .05973 | .118 | -.0297 | .2306 |
|  |  | PCM | .11999 | .05973 | .068 | -.0101 | .2501 |
|  | PSOM | PSONM | -.00777 | .05973 | .899 | -.1379 | .1224 |
|  |  | PCNM | .09268 | .05973 | .147 | -.0375 | .2228 |
|  |  | PCM | .11222 | .05973 | .085 | -.0179 | .2424 |
|  | PCNM | PSONM | -.10045 | .05973 | .118 | -.2306 | .0297 |
|  |  | PSOM | -.09268 | .05973 | .147 | -.2228 | .0375 |
|  |  | PCM | .01954 | .05973 | .749 | -.1106 | .1497 |
|  | PCM | PSONM | -.11999 | .05973 | .068 | -.2501 | .0101 |
|  |  | PSOM | -.11222 | .05973 | .085 | -.2424 | .0179 |
|  |  | PCNM | -.01954 | .05973 | .749 | -.1497 | .1106 |
| Firmicutes | PSONM | PSOM | .00092 | .06512 | .989 | -.1410 | .1428 |
|  |  | PCNM | -.08116 | .06512 | .236 | -.2230 | .0607 |
|  |  | PCM | -.12432 | .06512 | .080 | -.2662 | .0176 |
|  | PSOM | PSONM | -.00092 | .06512 | .989 | -.1428 | .1410 |
|  |  | PCNM | -.08207 | .06512 | .232 | -.2240 | .0598 |
|  |  | PCM | -.12524 | .06512 | .079 | -.2671 | .0166 |
|  | PCNM | PSONM | .08116 | .06512 | .236 | -.0607 | .2230 |
|  |  | PSOM | .08207 | .06512 | .232 | -.0598 | .2240 |
|  |  | PCM | -.04317 | .06512 | .520 | -.1851 | .0987 |
|  | PCM | PSONM | .12432 | .06512 | .080 | -.0176 | .2662 |
|  |  | PSOM | .12524 | .06512 | .079 | -.0166 | .2671 |
|  |  | PCNM | .04317 | .06512 | .520 | -.0987 | .1851 |
| Acidobacteria | PSONM | PSOM | -.00059 | .00130 | .658 | -.0034 | .0022 |
|  |  | PCNM | -.00235 | .00130 | .096 | -.0052 | .0005 |
|  |  | PCM | -.00447^*^ | .00130 | .005 | -.0073 | -.0016 |
|  | PSOM | PSONM | .00059 | .00130 | .658 | -.0022 | .0034 |
|  |  | PCNM | -.00176 | .00130 | .201 | -.0046 | .0011 |
|  |  | PCM | -.00388^*^ | .00130 | .011 | -.0067 | -.0011 |
|  | PCNM | PSONM | .00235 | .00130 | .096 | -.0005 | .0052 |
|  |  | PSOM | .00176 | .00130 | .201 | -.0011 | .0046 |
|  |  | PCM | -.00212 | .00130 | .128 | -.0050 | .0007 |
|  | PCM | PSONM | .00447^*^ | .00130 | .005 | .0016 | .0073 |
|  |  | PSOM | .00388^*^ | .00130 | .011 | .0011 | .0067 |
|  |  | PCNM | .00212 | .00130 | .128 | -.0007 | .0050 |
| Crenarchaeota | PSONM | PSOM | -.00024 | .00131 | .855 | -.0031 | .0026 |
|  |  | PCNM | -.00201 | .00131 | .151 | -.0049 | .0008 |
|  |  | PCM | -.00446^*^ | .00131 | .005 | -.0073 | -.0016 |
|  | PSOM | PSONM | .00024 | .00131 | .855 | -.0026 | .0031 |
|  |  | PCNM | -.00177 | .00131 | .202 | -.0046 | .0011 |
|  |  | PCM | -.00422^*^ | .00131 | .007 | -.0071 | -.0014 |
|  | PCNM | PSONM | .00201 | .00131 | .151 | -.0008 | .0049 |
|  |  | PSOM | .00177 | .00131 | .202 | -.0011 | .0046 |
|  |  | PCM | -.00245 | .00131 | .085 | -.0053 | .0004 |
|  | PCM | PSONM | .00446^*^ | .00131 | .005 | .0016 | .0073 |
|  |  | PSOM | .00422^*^ | .00131 | .007 | .0014 | .0071 |
|  |  | PCNM | .00245 | .00131 | .085 | -.0004 | .0053 |
| Actinobacteria | PSONM | PSOM | .05128^*^ | .02135 | .033 | .0048 | .0978 |
|  |  | PCNM | -.00078 | .02135 | .971 | -.0473 | .0457 |
|  |  | PCM | .04481 | .02135 | .058 | -.0017 | .0913 |
|  | PSOM | PSONM | -.05128^*^ | .02135 | .033 | -.0978 | -.0048 |
|  |  | PCNM | -.05206^*^ | .02135 | .031 | -.0986 | -.0055 |
|  |  | PCM | -.00647 | .02135 | .767 | -.0530 | .0401 |
|  | PCNM | PSONM | .00078 | .02135 | .971 | -.0457 | .0473 |
|  |  | PSOM | .05206^*^ | .02135 | .031 | .0055 | .0986 |
|  |  | PCM | .04560 | .02135 | .054 | -.0009 | .0921 |
|  | PCM | PSONM | -.04481 | .02135 | .058 | -.0913 | .0017 |
|  |  | PSOM | .00647 | .02135 | .767 | -.0401 | .0530 |
|  |  | PCNM | -.04560 | .02135 | .054 | -.0921 | .0009 |
| Chloroflexi | PSONM | PSOM | -.00051 | .00195 | .796 | -.0048 | .0037 |
|  |  | PCNM | -.00412 | .00195 | .056 | -.0084 | .0001 |
|  |  | PCM | -.00608^*^ | .00195 | .009 | -.0103 | -.0018 |
|  | PSOM | PSONM | .00051 | .00195 | .796 | -.0037 | .0048 |
|  |  | PCNM | -.00361 | .00195 | .089 | -.0079 | .0006 |
|  |  | PCM | -.00556^*^ | .00195 | .015 | -.0098 | -.0013 |
|  | PCNM | PSONM | .00412 | .00195 | .056 | -.0001 | .0084 |
|  |  | PSOM | .00361 | .00195 | .089 | -.0006 | .0079 |
|  |  | PCM | -.00195 | .00195 | .336 | -.0062 | .0023 |
|  | PCM | PSONM | .00608^*^ | .00195 | .009 | .0018 | .0103 |
|  |  | PSOM | .00556^*^ | .00195 | .015 | .0013 | .0098 |
|  |  | PCNM | .00195 | .00195 | .336 | -.0023 | .0062 |
| Bacteroidetes | PSONM | PSOM | -.04682 | .02477 | .083 | -.1008 | .0072 |
|  |  | PCNM | -.00460 | .02477 | .856 | -.0586 | .0494 |
|  |  | PCM | -.00117 | .02477 | .963 | -.0551 | .0528 |
|  | PSOM | PSONM | .04682 | .02477 | .083 | -.0072 | .1008 |
|  |  | PCNM | .04222 | .02477 | .114 | -.0118 | .0962 |
|  |  | PCM | .04564 | .02477 | .090 | -.0083 | .0996 |
|  | PCNM | PSONM | .00460 | .02477 | .856 | -.0494 | .0586 |
|  |  | PSOM | -.04222 | .02477 | .114 | -.0962 | .0118 |
|  |  | PCM | .00342 | .02477 | .892 | -.0506 | .0574 |
|  | PCM | PSONM | .00117 | .02477 | .963 | -.0528 | .0551 |
|  |  | PSOM | -.04564 | .02477 | .090 | -.0996 | .0083 |
|  |  | PCNM | -.00342 | .02477 | .892 | -.0574 | .0506 |
| Planctomycetes | PSONM | PSOM | -.00023 | .00091 | .808 | -.0022 | .0018 |
|  |  | PCNM | -.00360^*^ | .00091 | .002 | -.0056 | -.0016 |
|  |  | PCM | -.00420^*^ | .00091 | .001 | -.0062 | -.0022 |
|  | PSOM | PSONM | .00023 | .00091 | .808 | -.0018 | .0022 |
|  |  | PCNM | -.00337^*^ | .00091 | .003 | -.0054 | -.0014 |
|  |  | PCM | -.00398^*^ | .00091 | .001 | -.0060 | -.0020 |
|  | PCNM | PSONM | .00360^*^ | .00091 | .002 | .0016 | .0056 |
|  |  | PSOM | .00337^*^ | .00091 | .003 | .0014 | .0054 |
|  |  | PCM | -.00060 | .00091 | .520 | -.0026 | .0014 |
|  | PCM | PSONM | .00420^*^ | .00091 | .001 | .0022 | .0062 |
|  |  | PSOM | .00398^*^ | .00091 | .001 | .0020 | .0060 |
|  |  | PCNM | .00060 | .00091 | .520 | -.0014 | .0026 |
| Nitrospirae | PSONM | PSOM | -.00043 | .00127 | .739 | -.0032 | .0023 |
|  |  | PCNM | -.00183 | .00127 | .176 | -.0046 | .0009 |
|  |  | PCM | -.00415^*^ | .00127 | .007 | -.0069 | -.0014 |
|  | PSOM | PSONM | .00043 | .00127 | .739 | -.0023 | .0032 |
|  |  | PCNM | -.00139 | .00127 | .295 | -.0042 | .0014 |
|  |  | PCM | -.00372^*^ | .00127 | .013 | -.0065 | -.0009 |
|  | PCNM | PSONM | .00183 | .00127 | .176 | -.0009 | .0046 |
|  |  | PSOM | .00139 | .00127 | .295 | -.0014 | .0042 |
|  |  | PCM | -.00232 | .00127 | .092 | -.0051 | .0004 |
|  | PCM | PSONM | .00415^*^ | .00127 | .007 | .0014 | .0069 |
|  |  | PSOM | .00372^*^ | .00127 | .013 | .0009 | .0065 |
|  |  | PCNM | .00232 | .00127 | .092 | -.0004 | .0051 |
| Cyanobacteria | PSONM | PSOM | -.00046 | .00529 | .932 | -.0120 | .0111 |
|  |  | PCNM | -.00698 | .00529 | .212 | -.0185 | .0046 |
|  |  | PCM | -.01268^*^ | .00529 | .034 | -.0242 | -.0011 |
|  | PSOM | PSONM | .00046 | .00529 | .932 | -.0111 | .0120 |
|  |  | PCNM | -.00652 | .00529 | .242 | -.0181 | .0050 |
|  |  | PCM | -.01222^*^ | .00529 | .040 | -.0237 | -.0007 |
|  | PCNM | PSONM | .00698 | .00529 | .212 | -.0046 | .0185 |
|  |  | PSOM | .00652 | .00529 | .242 | -.0050 | .0181 |
|  |  | PCM | -.00570 | .00529 | .303 | -.0172 | .0058 |
|  | PCM | PSONM | .01268^*^ | .00529 | .034 | .0011 | .0242 |
|  |  | PSOM | .01222^*^ | .00529 | .040 | .0007 | .0237 |
|  |  | PCNM | .00570 | .00529 | .303 | -.0058 | .0172 |
| Other | PSONM | PSOM | -.00994 | .00496 | .068 | -.0207 | .0009 |
|  |  | PCNM | .01056 | .00496 | .055 | -.0002 | .0214 |
|  |  | PCM | .00122 | .00496 | .810 | -.0096 | .0120 |
|  | PSOM | PSONM | .00994 | .00496 | .068 | -.0009 | .0207 |
|  |  | PCNM | .02050^*^ | .00496 | .001 | .0097 | .0313 |
|  |  | PCM | .01116^*^ | .00496 | .044 | .0003 | .0220 |
|  | PCNM | PSONM | -.01056 | .00496 | .055 | -.0214 | .0002 |
|  |  | PSOM | -.02050^*^ | .00496 | .001 | -.0313 | -.0097 |
|  |  | PCM | -.00934 | .00496 | .084 | -.0202 | .0015 |
|  | PCM | PSONM | -.00122 | .00496 | .810 | -.0120 | .0096 |
|  |  | PSOM | -.01116^*^ | .00496 | .044 | -.0220 | -.0003 |
|  |  | PCNM | .00934 | .00496 | .084 | -.0015 | .0202 |
| *. The mean difference is significant at the 0.05 level. | | | | | | | |

Table S3. The analysis of variance among soil, phyllosphere and air samples (family level).

|  | | | Mean Difference (I-J) | Std.Error | Sig. | 95% Confidence Interval | |
| --- | --- | --- | --- | --- | --- | --- | --- |
|  |  |  |  |  |  | Lower Bound | Upper Bound |
|  |  |  |  |  |  |  |  |
| Bacillaceae | Soil | Phyllosphere | -.03412 | .02297 | .146 | -.0807 | .0125 |
|  |  | Air | .02936 | .03110 | .352 | -.0338 | .0925 |
|  | Phyllosphere | Soil | .03412 | .02297 | .146 | -.0125 | .0807 |
|  |  | Air | .06348^*^ | .03110 | .049 | .0003 | .1266 |
| Nitrososphaeraceae | Soil | Phyllosphere | .12142^*^ | .01119 | .000 | .0987 | .1441 |
|  |  | Air | .05027^*^ | .01515 | .002 | .0195 | .0810 |
|  | Phyllosphere | Soil | -.12142^*^ | .01119 | .000 | -.1441 | -.0987 |
|  |  | Air | -.07115^*^ | .01515 | .000 | -.1019 | -.0404 |
| Rhizobiaceae | Soil | Phyllosphere | -.37361^*^ | .04071 | .000 | -.4563 | -.2910 |
|  |  | Air | -.00019 | .05512 | .997 | -.1121 | .1117 |
|  | Phyllosphere | Soil | .37361^*^ | .04071 | .000 | .2910 | .4563 |
|  |  | Air | .37342^*^ | .05512 | .000 | .2615 | .4853 |
| Pseudomonadaceae | Soil | Phyllosphere | -.13899^*^ | .03141 | .000 | -.2027 | -.0752 |
|  |  | Air | -.00686 | .04252 | .873 | -.0932 | .0795 |
|  | Phyllosphere | Soil | .13899^*^ | .03141 | .000 | .0752 | .2027 |
|  |  | Air | .13212^*^ | .04252 | .004 | .0458 | .2185 |
| Hyphomicrobiaceae | Soil | Phyllosphere | .02248^*^ | .00320 | .000 | .0160 | .0290 |
|  |  | Air | .01427^*^ | .00433 | .002 | .0055 | .0231 |
|  | Phyllosphere | Soil | -.02248^*^ | .00320 | .000 | -.0290 | -.0160 |
|  |  | Air | -.00821 | .00433 | .066 | -.0170 | .0006 |
| Pirellulaceae | Soil | Phyllosphere | .02993^*^ | .00110 | .000 | .0277 | .0322 |
|  |  | Air | .00530^*^ | .00149 | .001 | .0023 | .0083 |
|  | Phyllosphere | Soil | -.02993^*^ | .00110 | .000 | -.0322 | -.0277 |
|  |  | Air | -.02462^*^ | .00149 | .000 | -.0276 | -.0216 |
| Syntrophobacteraceae | Soil | Phyllosphere | .02609^*^ | .00085 | .000 | .0244 | .0278 |
|  |  | Air | -.00396^*^ | .00115 | .002 | -.0063 | -.0016 |
|  | Phyllosphere | Soil | -.02609^*^ | .00085 | .000 | -.0278 | -.0244 |
|  |  | Air | -.03005^*^ | .00115 | .000 | -.0324 | -.0277 |
| Chitinophagaceae | Soil | Phyllosphere | .01961^*^ | .00158 | .000 | .0164 | .0228 |
|  |  | Air | .01904^*^ | .00213 | .000 | .0147 | .0234 |
|  | Phyllosphere | Soil | -.01961^*^ | .00158 | .000 | -.0228 | -.0164 |
|  |  | Air | -.00057 | .00213 | .792 | -.0049 | .0038 |
| Cytophagaceae | Soil | Phyllosphere | .01950^*^ | .00176 | .000 | .0159 | .0231 |
|  |  | Air | .01948^*^ | .00239 | .000 | .0146 | .0243 |
|  | Phyllosphere | Soil | -.01950^*^ | .00176 | .000 | -.0231 | -.0159 |
|  |  | Air | -.00002 | .00239 | .994 | -.0049 | .0048 |
| Xanthomonadaceae | Soil | Phyllosphere | -.02744^*^ | .01063 | .014 | -.0490 | -.0059 |
|  |  | Air | -.00154 | .01439 | .916 | -.0308 | .0277 |
|  | Phyllosphere | Soil | .02744^*^ | .01063 | .014 | .0059 | .0490 |
|  |  | Air | .02590 | .01439 | .081 | -.0033 | .0551 |
| Other | Soil | Phyllosphere | .01745^*^ | .00260 | .000 | .0122 | .0227 |
|  |  | Air | -.00882^*^ | .00351 | .017 | -.0159 | -.0017 |
|  | Phyllosphere | Soil | -.01745^*^ | .00260 | .000 | -.0227 | -.0122 |
|  |  | Air | -.02626^*^ | .00351 | .000 | -.0334 | -.0191 |
| *. The mean difference is significant at the 0.05 level. | | | | | | | |

Table S4. The analysis of variance between plant species in different chamber (family level).

|  | | | Mean Difference (I-J) | Std.Error | Sig. | 95% Confidence Interval | |
| --- | --- | --- | --- | --- | --- | --- | --- |
|  |  |  |  |  |  | Lower Bound | Upper Bound |
|  |  |  |  |  |  |  |  |
| Rhizobiaceae | PSONM | PSOM | .03003 | .11882 | .805 | -.2289 | .2889 |
|  |  | PCNM | .10790 | .11882 | .382 | -.1510 | .3668 |
|  |  | PCM | .22889 | .11882 | .078 | -.0300 | .4878 |
|  | PSOM | PSONM | -.03003 | .11882 | .805 | -.2889 | .2289 |
|  |  | PCNM | .07787 | .11882 | .525 | -.1810 | .3368 |
|  |  | PCM | .19886 | .11882 | .120 | -.0600 | .4578 |
|  | PCNM | PSONM | -.10790 | .11882 | .382 | -.3668 | .1510 |
|  |  | PSOM | -.07787 | .11882 | .525 | -.3368 | .1810 |
|  |  | PCM | .12099 | .11882 | .329 | -.1379 | .3799 |
|  | PCM | PSONM | -.22889 | .11882 | .078 | -.4878 | .0300 |
|  |  | PSOM | -.19886 | .11882 | .120 | -.4578 | .0600 |
|  |  | PCNM | -.12099 | .11882 | .329 | -.3799 | .1379 |
| Nitrososphaeraceae | PSONM | PSOM | -.00019 | .00106 | .859 | -.0025 | .0021 |
|  |  | PCNM | -.00205 | .00106 | .077 | -.0044 | .0003 |
|  |  | PCM | -.00366^*^ | .00106 | .005 | -.0060 | -.0014 |
|  | PSOM | PSONM | .00019 | .00106 | .859 | -.0021 | .0025 |
|  |  | PCNM | -.00186 | .00106 | .105 | -.0042 | .0004 |
|  |  | PCM | -.00347^*^ | .00106 | .007 | -.0058 | -.0012 |
|  | PCNM | PSONM | .00205 | .00106 | .077 | -.0003 | .0044 |
|  |  | PSOM | .00186 | .00106 | .105 | -.0004 | .0042 |
|  |  | PCM | -.00161 | .00106 | .155 | -.0039 | .0007 |
|  | PCM | PSONM | .00366^*^ | .00106 | .005 | .0014 | .0060 |
|  |  | PSOM | .00347^*^ | .00106 | .007 | .0012 | .0058 |
|  |  | PCNM | .00161 | .00106 | .155 | -.0007 | .0039 |
| Bacillaceae | PSONM | PSOM | .06730 | .05606 | .253 | -.0548 | .1894 |
|  |  | PCNM | -.06644 | .05606 | .259 | -.1886 | .0557 |
|  |  | PCM | -.10858 | .05606 | .077 | -.2307 | .0136 |
|  | PSOM | PSONM | -.06730 | .05606 | .253 | -.1894 | .0548 |
|  |  | PCNM | -.13374^*^ | .05606 | .034 | -.2559 | -.0116 |
|  |  | PCM | -.17588^*^ | .05606 | .009 | -.2980 | -.0537 |
|  | PCNM | PSONM | .06644 | .05606 | .259 | -.0557 | .1886 |
|  |  | PSOM | .13374^*^ | .05606 | .034 | .0116 | .2559 |
|  |  | PCM | -.04214 | .05606 | .467 | -.1643 | .0800 |
|  | PCM | PSONM | .10858 | .05606 | .077 | -.0136 | .2307 |
|  |  | PSOM | .17588^*^ | .05606 | .009 | .0537 | .2980 |
|  |  | PCNM | .04214 | .05606 | .467 | -.0800 | .1643 |
| Pseudomonadaceae | PSONM | PSOM | -.06763 | .08393 | .436 | -.2505 | .1152 |
|  |  | PCNM | .15626 | .08393 | .087 | -.0266 | .3391 |
|  |  | PCM | .00656 | .08393 | .939 | -.1763 | .1894 |
|  | PSOM | PSONM | .06763 | .08393 | .436 | -.1152 | .2505 |
|  |  | PCNM | .22390^*^ | .08393 | .020 | .0410 | .4068 |
|  |  | PCM | .07420 | .08393 | .394 | -.1087 | .2571 |
|  | PCNM | PSONM | -.15626 | .08393 | .087 | -.3391 | .0266 |
|  |  | PSOM | -.22390^*^ | .08393 | .020 | -.4068 | -.0410 |
|  |  | PCM | -.14970 | .08393 | .100 | -.3326 | .0332 |
|  | PCM | PSONM | -.00656 | .08393 | .939 | -.1894 | .1763 |
|  |  | PSOM | -.07420 | .08393 | .394 | -.2571 | .1087 |
|  |  | PCNM | .14970 | .08393 | .100 | -.0332 | .3326 |
| Hyphomicrobiaceae | PSONM | PSOM | .00282 | .00580 | .635 | -.0098 | .0155 |
|  |  | PCNM | -.02384^*^ | .00580 | .001 | -.0365 | -.0112 |
|  |  | PCM | -.00615 | .00580 | .310 | -.0188 | .0065 |
|  | PSOM | PSONM | -.00282 | .00580 | .635 | -.0155 | .0098 |
|  |  | PCNM | -.02666^*^ | .00580 | .001 | -.0393 | -.0140 |
|  |  | PCM | -.00897 | .00580 | .148 | -.0216 | .0037 |
|  | PCNM | PSONM | .02384^*^ | .00580 | .001 | .0112 | .0365 |
|  |  | PSOM | .02666^*^ | .00580 | .001 | .0140 | .0393 |
|  |  | PCM | .01769^*^ | .00580 | .010 | .0051 | .0303 |
|  | PCM | PSONM | .00615 | .00580 | .310 | -.0065 | .0188 |
|  |  | PSOM | .00897 | .00580 | .148 | -.0037 | .0216 |
|  |  | PCNM | -.01769^*^ | .00580 | .010 | -.0303 | -.0051 |
| Pirellulaceae | PSONM | PSOM | -.00014 | .00054 | .802 | -.0013 | .0010 |
|  |  | PCNM | -.00123^*^ | .00054 | .041 | -.0024 | -.0001 |
|  |  | PCM | -.00218^*^ | .00054 | .002 | -.0033 | -.0010 |
|  | PSOM | PSONM | .00014 | .00054 | .802 | -.0010 | .0013 |
|  |  | PCNM | -.00109 | .00054 | .065 | -.0023 | .0001 |
|  |  | PCM | -.00204^*^ | .00054 | .003 | -.0032 | -.0009 |
|  | PCNM | PSONM | .00123^*^ | .00054 | .041 | .0001 | .0024 |
|  |  | PSOM | .00109 | .00054 | .065 | -.0001 | .0023 |
|  |  | PCM | -.00095 | .00054 | .102 | -.0021 | .0002 |
|  | PCM | PSONM | .00218^*^ | .00054 | .002 | .0010 | .0033 |
|  |  | PSOM | .00204^*^ | .00054 | .003 | .0009 | .0032 |
|  |  | PCNM | .00095 | .00054 | .102 | -.0002 | .0021 |
| Syntrophobacteraceae | PSONM | PSOM | -.00014 | .00048 | .783 | -.0012 | .0009 |
|  |  | PCNM | -.00109^*^ | .00048 | .043 | -.0021 | .0000 |
|  |  | PCM | -.00152^*^ | .00048 | .008 | -.0026 | -.0005 |
|  | PSOM | PSONM | .00014 | .00048 | .783 | -.0009 | .0012 |
|  |  | PCNM | -.00095 | .00048 | .072 | -.0020 | .0001 |
|  |  | PCM | -.00139^*^ | .00048 | .014 | -.0024 | -.0003 |
|  | PCNM | PSONM | .00109^*^ | .00048 | .043 | .0000 | .0021 |
|  |  | PSOM | .00095 | .00048 | .072 | -.0001 | .0020 |
|  |  | PCM | -.00043 | .00048 | .387 | -.0015 | .0006 |
|  | PCM | PSONM | .00152^*^ | .00048 | .008 | .0005 | .0026 |
|  |  | PSOM | .00139^*^ | .00048 | .014 | .0003 | .0024 |
|  |  | PCNM | .00043 | .00048 | .387 | -.0006 | .0015 |
| Chitinophagaceae | PSONM | PSOM | -.00068 | .00056 | .246 | -.0019 | .0005 |
|  |  | PCNM | -.01399^*^ | .00056 | .000 | -.0152 | -.0128 |
|  |  | PCM | -.00058 | .00056 | .321 | -.0018 | .0006 |
|  | PSOM | PSONM | .00068 | .00056 | .246 | -.0005 | .0019 |
|  |  | PCNM | -.01331^*^ | .00056 | .000 | -.0145 | -.0121 |
|  |  | PCM | .00010 | .00056 | .856 | -.0011 | .0013 |
|  | PCNM | PSONM | .01399^*^ | .00056 | .000 | .0128 | .0152 |
|  |  | PSOM | .01331^*^ | .00056 | .000 | .0121 | .0145 |
|  |  | PCM | .01341^*^ | .00056 | .000 | .0122 | .0146 |
|  | PCM | PSONM | .00058 | .00056 | .321 | -.0006 | .0018 |
|  |  | PSOM | -.00010 | .00056 | .856 | -.0013 | .0011 |
|  |  | PCNM | -.01341^*^ | .00056 | .000 | -.0146 | -.0122 |
| Cytophagaceae | PSONM | PSOM | -.00067 | .00432 | .879 | -.0101 | .0088 |
|  |  | PCNM | -.00442 | .00432 | .327 | -.0138 | .0050 |
|  |  | PCM | -.00874 | .00432 | .066 | -.0182 | .0007 |
|  | PSOM | PSONM | .00067 | .00432 | .879 | -.0088 | .0101 |
|  |  | PCNM | -.00375 | .00432 | .403 | -.0132 | .0057 |
|  |  | PCM | -.00807 | .00432 | .087 | -.0175 | .0014 |
|  | PCNM | PSONM | .00442 | .00432 | .327 | -.0050 | .0138 |
|  |  | PSOM | .00375 | .00432 | .403 | -.0057 | .0132 |
|  |  | PCM | -.00432 | .00432 | .337 | -.0137 | .0051 |
|  | PCM | PSONM | .00874 | .00432 | .066 | -.0007 | .0182 |
|  |  | PSOM | .00807 | .00432 | .087 | -.0014 | .0175 |
|  |  | PCNM | .00432 | .00432 | .337 | -.0051 | .0137 |
| Xanthomonadaceae | PSONM | PSOM | .00506 | .03106 | .873 | -.0626 | .0727 |
|  |  | PCNM | .02531 | .03106 | .431 | -.0424 | .0930 |
|  |  | PCM | -.03829 | .03106 | .241 | -.1060 | .0294 |
|  | PSOM | PSONM | -.00506 | .03106 | .873 | -.0727 | .0626 |
|  |  | PCNM | .02025 | .03106 | .527 | -.0474 | .0879 |
|  |  | PCM | -.04336 | .03106 | .188 | -.1110 | .0243 |
|  | PCNM | PSONM | -.02531 | .03106 | .431 | -.0930 | .0424 |
|  |  | PSOM | -.02025 | .03106 | .527 | -.0879 | .0474 |
|  |  | PCM | -.06361 | .03106 | .063 | -.1313 | .0041 |
|  | PCM | PSONM | .03829 | .03106 | .241 | -.0294 | .1060 |
|  |  | PSOM | .04336 | .03106 | .188 | -.0243 | .1110 |
|  |  | PCNM | .06361 | .03106 | .063 | -.0041 | .1313 |
| Other | PSONM | PSOM | -.00994 | .00496 | .068 | -.0207 | .0009 |
|  |  | PCNM | .01060 | .00496 | .054 | -.0002 | .0214 |
|  |  | PCM | .00122 | .00496 | .810 | -.0096 | .0120 |
|  | PSOM | PSONM | .00994 | .00496 | .068 | -.0009 | .0207 |
|  |  | PCNM | .02054^*^ | .00496 | .001 | .0097 | .0314 |
|  |  | PCM | .01116^*^ | .00496 | .044 | .0003 | .0220 |
|  | PCNM | PSONM | -.01060 | .00496 | .054 | -.0214 | .0002 |
|  |  | PSOM | -.02054^*^ | .00496 | .001 | -.0314 | -.0097 |
|  |  | PCM | -.00938 | .00496 | .083 | -.0202 | .0014 |
|  | PCM | PSONM | -.00122 | .00496 | .810 | -.0120 | .0096 |
|  |  | PSOM | -.01116^*^ | .00496 | .044 | -.0220 | -.0003 |
|  |  | PCNM | .00938 | .00496 | .083 | -.0014 | .0202 |
| *. The mean difference is significant at the 0.05 level. | | | | | | | |


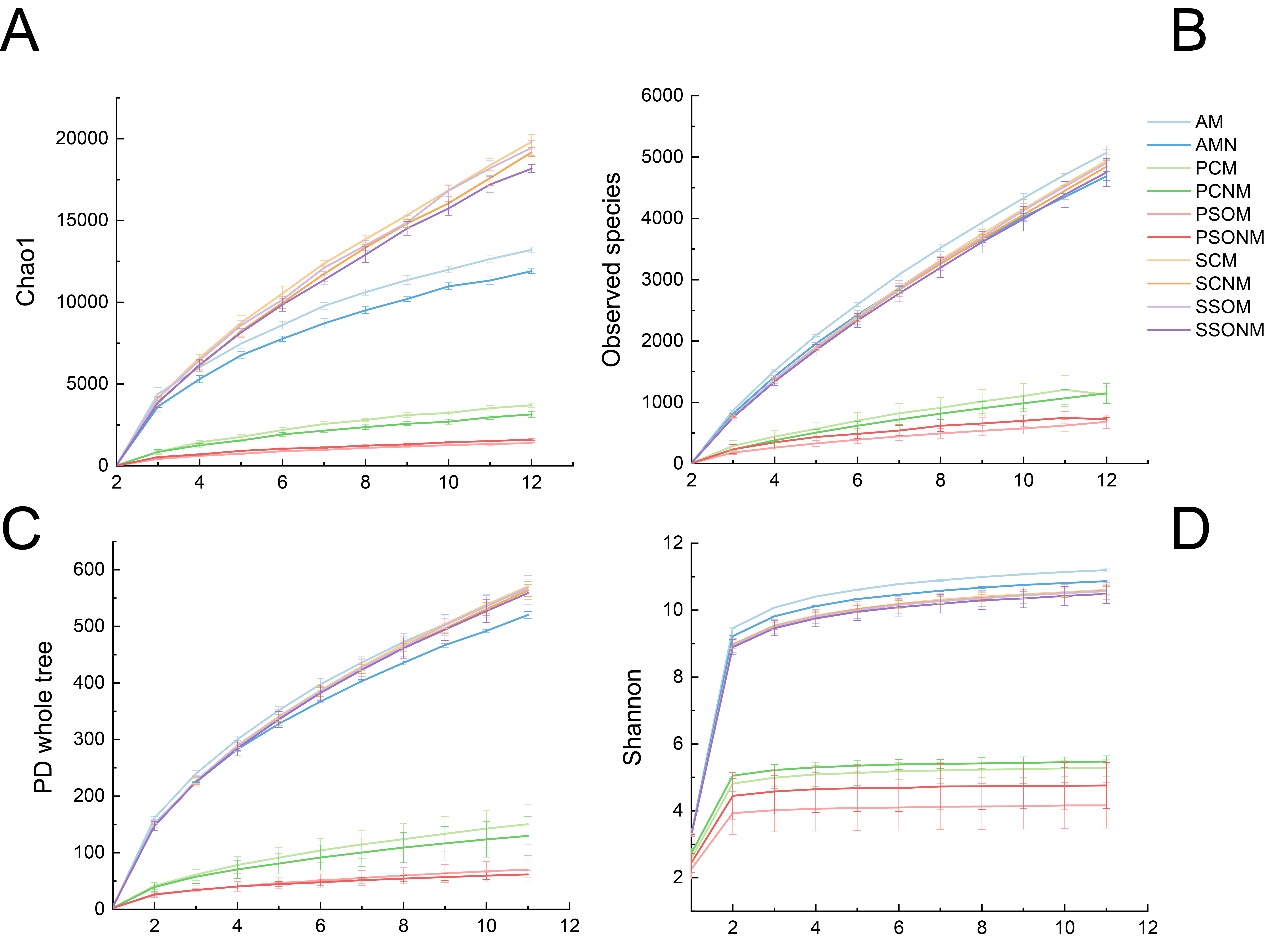


**Figure S2.** Chao1, Observed species, PD whole tree and Shannon analysis of Alpha diversity. “M” represents chamber 1 samples (with extra outdoor microbes) and “NM” represents chamber 2 samples (without extra microbes). AM, PCM, PSOM, SCM and SSOM represent air, phyllosphere and soil of *Allium schoenoprasum* and *Sonchus oleraceus* samples, respectively in chamber 1 (with external airborne microbes). ANM, PCNM, PSONM, SCNM and SSONM represent the air, phyllosphere and soil of *A. schoenoprasum* and *S. oleraceus* samples, respectively in chamber 2 (without external airborne microbes).


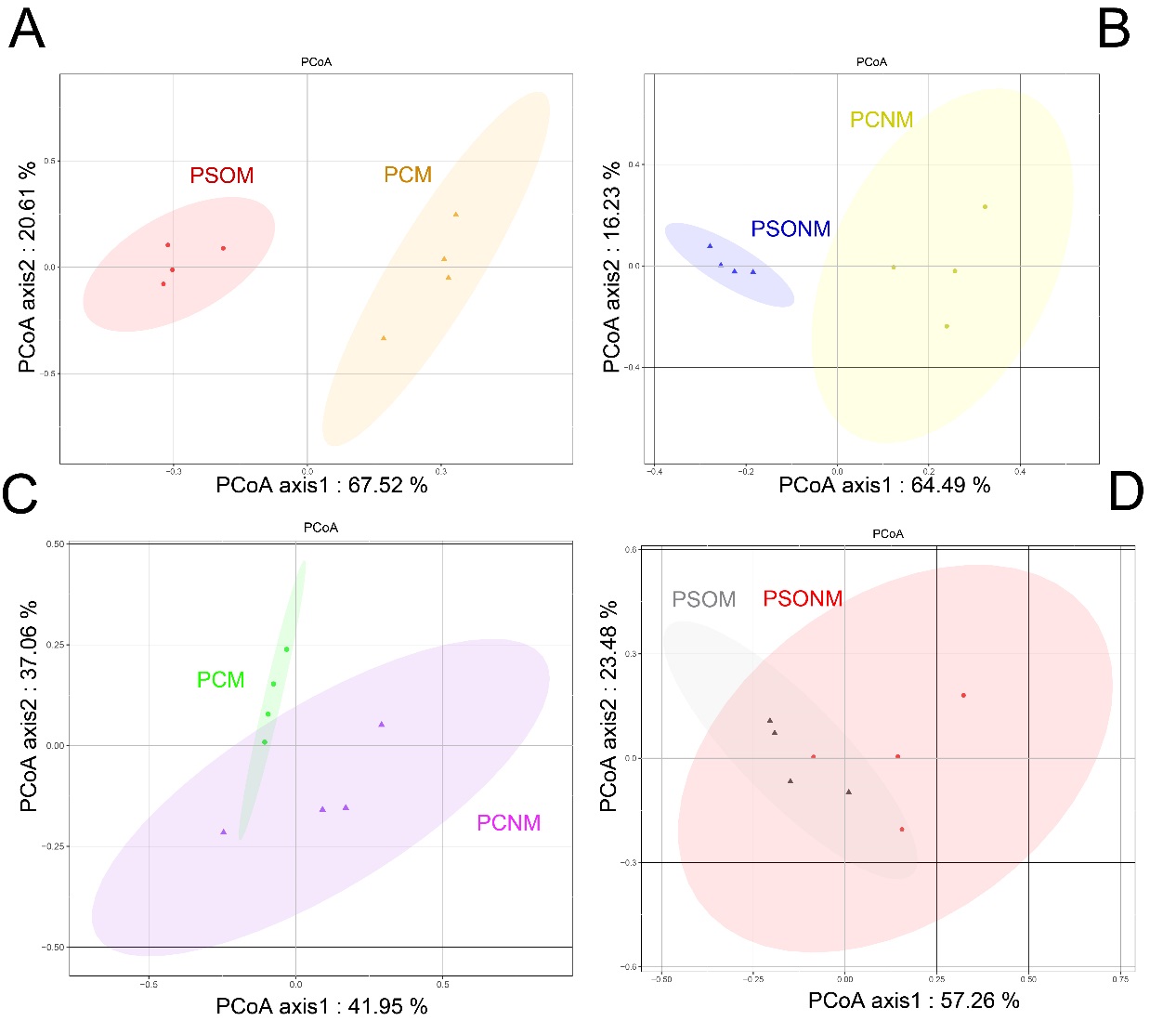


**Figure S3.** Principal Coordinate Analysis (PCoA) based on Bray-Curtis distances of bacterial OTU. (A) PCoA between *Allium schoenoprasum* and *Sonchus oleraceus* samples in chamber 1 (P < 0.05, PERMANOVA). PCM and PSOM represent *A. schoenoprasum* and *S. oleraceus* samples in chamber 1 (with extra air microbes), respectively. (B) PCoA between *A. schoenoprasum* and *S. oleraceus* samples in chamber 2 (P < 0.05, PERMANOVA). The PCNM and PSONM represent *A. schoenoprasum* and *S. oleraceus* samples in chamber 2 (without extra air microbes), respectively. (C) and (D) PCoA of *A. schoenoprasum* (P < 0.05, PERMANOVA) and *S. oleraceus* (P > 0.05, PERMANOVA) in chamber 1 and 2.
